# NLRP3 inflammasome activation in neutrophils directs early inflammatory response in murine peritonitis

**DOI:** 10.1038/s41598-022-25176-4

**Published:** 2022-12-09

**Authors:** Saeko Fukui, Shoichi Fukui, Stijn Van Bruggen, Lai Shi, Casey E. Sheehy, Long Chu, Denisa D. Wagner

**Affiliations:** 1 Program in Cellular and Molecular Medicine, Boston Children’s Hospital, Boston, MA 02115 USA; 2 Department of Pediatrics, Harvard Medical School, Boston, MA 02115 USA; 3 Division of Hematology/Oncology, Boston Children’s Hospital, Boston, MA 02115 USA; 4 Center for Molecular and Vascular Biology, Department of Cardiovascular Sciences, KU Leuven, 3000 Leuven, Belgium

**Keywords:** Immunology, Molecular biology

## Abstract

NLR family pyrin domain containing 3 (NLRP3) inflammasome mediates caspase-1-dependent processing of inflammatory cytokines such as IL-1β, an essential endothelial activator, and contributes to the pathology of inflammatory diseases. To evaluate the role of NLRP3 in neutrophils in endothelial activation, which is still elusive, we used the thioglycollate-induced peritonitis model characterized by an early neutrophil influx, on *Nlrp3*^*−*/*−*^ and *Nlrp3*^+/+^ mice. *Nlrp3*^*−/−*^ mice recruited fewer neutrophils than *Nlrp3*^+/+^ into the peritoneum and showed lower IL-1β in peritoneal lavage fluid. The higher production of IL-1β in *Nlrp3*^+/+^ was neutrophil-dependent as neutrophil depletion prevented the IL-1β production. The *Nlrp3*^+/+^ neutrophils collected from the peritoneal fluid formed significantly more filaments (specks) than *Nlrp3*^*−*/*−*^ neutrophils of ASC (apoptosis-associated speck-like protein containing a caspase activating and recruitment domain), a readout for inflammasome activation. Intravital microscopy revealed that leukocytes rolled significantly slower in *Nlrp3*^+/+^ venules than in *Nlrp3*^*−*/*−*^. *Nlrp3*^*−*/*−*^ endothelial cells isolated from mesenteric vessels demonstrated a lower percentage of P-selectin-positive cells with lower intensity of surface P-selectin expression than the *Nlrp3*^+/+^ endothelial cells evaluated by flow cytometry. We conclude that neutrophils orchestrate acute thioglycollate-induced peritonitis by producing IL-1β in an NLRP3-dependent manner. This increases endothelial P-selectin expression and leukocyte transmigration.

## Introduction

Inflammation is an important process safeguarding the host from infections and improving wound repair. Leukocytes circulating in blood vessels recognize that there is a problem in surrounding tissue by patrolling endothelial cells covering the walls of veins. The endothelium senses the injury or infection in its proximity and modifies its luminal plasma membrane to become sticky to leukocytes by expressing adhesion molecules, thus inducing leukocyte interaction with the vessel wall^1^. This process consists of multiple sequential steps: leukocyte tethering, rolling, adhesion, and transmigration, all enabled by the interaction of adhesion molecules exposed on the activated endothelial cells and their receptors on the surface of leukocytes. Leukocytes are captured and begin initial rolling on endothelial P-selectin, and its ligand on leukocytes, the P-selectin glycoprotein ligand-1 (PSGL-1). Leukocytes decrease their rolling velocity as inflammation progresses and more P-selectin is exposed on the endothelium. Leukocyte activation leads to firm adhesion of their integrins to upregulated endothelial cell adhesion molecules. Adherent leukocytes migrate through the microvasculature and complete the extravasation process to the inflamed site^1^.

Neutrophils are widely recognized as the first cells to be recruited to an inflammatory site. They play an essential role in an acute inflammatory reaction through several distinct mechanisms, such as phagocytosis, degranulation of bioactive molecules, and formation of neutrophil extracellular traps (NETs). While the role of the endothelium in neutrophil recruitment is well-defined as discussed above, whether transmigrating neutrophils further increase the endothelial adhesiveness during inflammation is not known. We found this question intriguing.

NLR family pyrin domain containing 3 (NLRP3) is an intracellular sensor that detects pathogen-associated molecular patterns (PAMPs) and damage-associated molecular patterns (DAMPs), leading to the formation of the NLRP3 inflammasome. Inflammasome mediates the production of inflammatory cytokines such as IL-1β, and regulates inflammatory responses and immune defense^2^.

The mechanisms of NLRP3 inflammasome activation in monocytes and macrophages have been extensively investigated. By contrast, the study of NLRP3 in neutrophils is still in its infancy, even though neutrophils are present at the site of inflammation and therefore represent a potential source of IL-1β. Recently, some studies reported the role of NLRP3 in the inflammatory reaction of neutrophils, including in neutrophil extracellular traps (NETs) formation^3^, in neutrophil recruitment in a mouse model of gout^4^, and in hepatic ischemia–reperfusion injury^5^. However, the role of NLRP3 in neutrophils is still elusive in inflammation regulation.

This study aims to elucidate whether neutrophils and *Nlrp3* regulate neutrophil-mediated inflammation. We focused mainly on the process of early neutrophil recruitment and the role of neutrophils in IL-1β generation by using a mouse thioglycollate-induced peritonitis model in the first 4 h. Here neutrophils are the predominant leukocyte recruited to the inflammatory site. We demonstrate by intravital microscopy that *Nlrp3* regulates leukocyte rolling on the vessel wall and neutrophil IL-1β production and neutrophil recruitment to the inflamed site via the activation of endothelial cells characterized by increased P-selectin expression.

## Results

**Figure 1:**
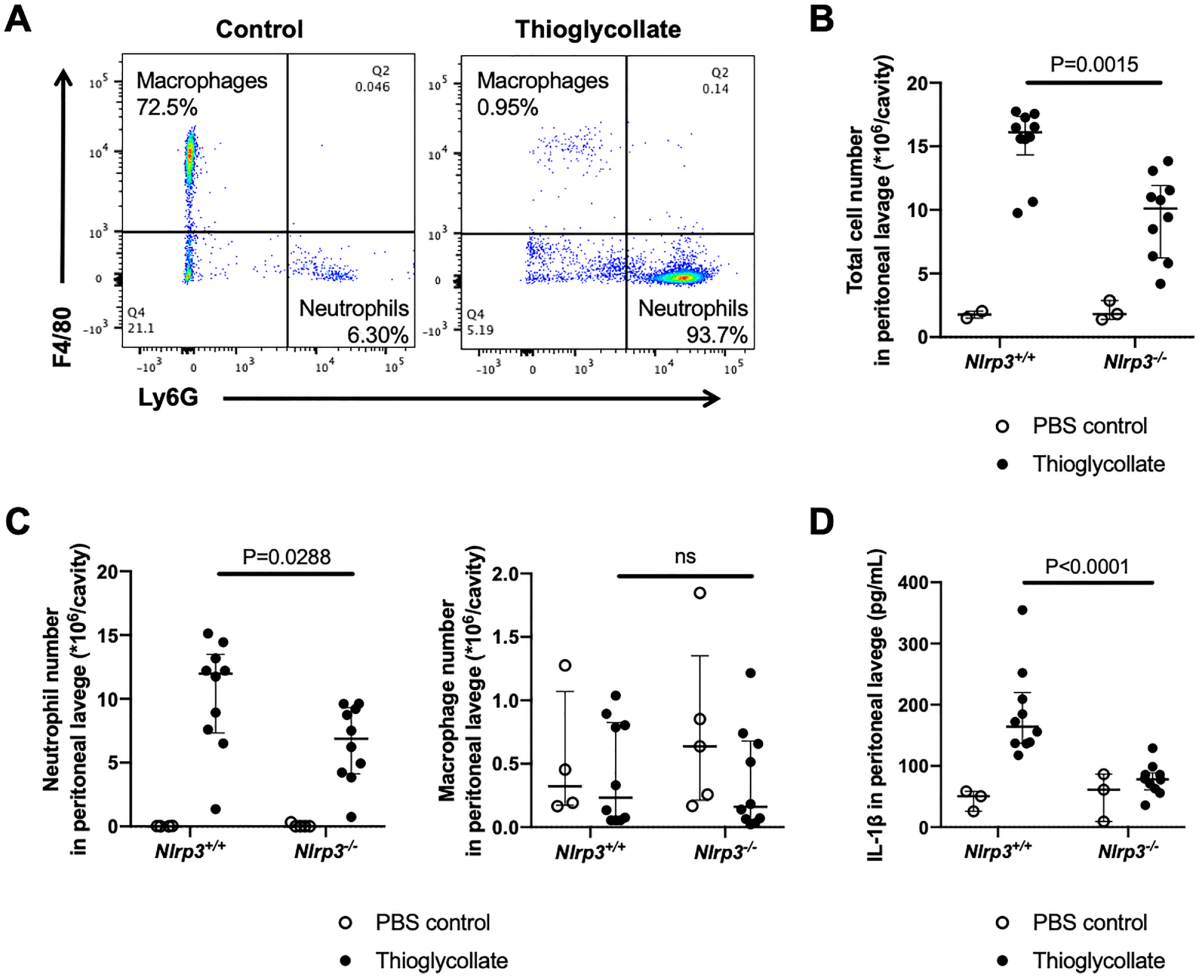
*Nlrp3*^*-/-*^ mice show less inflammatory cell recruitment and reduced levels of IL-1β in the peritoneal lavage fluid in the thioglycollate-induced peritonitis model. Mice were injected intraperitoneally (i.p.) with 1 mL sterile 3% thioglycollate broth (closed circles) or 1 mL sterile PBS (open circles). Four hours afterward, intraperitoneal cells were collected, counted, and analyzed for neutrophils and macrophages by flow cytometry. In addition, peritoneal lavage fluid was collected for cytokine measurements. (**A**) Neutrophils and macrophages in the intraperitoneal lavage fluid were defined as Ly6G-positive cells and F4/80-positive cells, respectively. (**B**) The total cell number in the peritoneal lavage fluid of *Nlrp3*^+*/*+^ and *Nlrp3*^*−/−*^ (PBS control, n = 3:3; Thioglycollate, n = 10:10). (**C**) The absolute numbers of neutrophils and macrophages in the peritoneal lavage fluid analyzed by flow cytometry (PBS control, n = 4:5; Thioglycollate, n = 10:10). (**D**) IL-1β in peritoneal lavage fluid measured by ELISA (PBS control, n = 3:3; Thioglycollate, n = 10:10).

**Figure 2:**
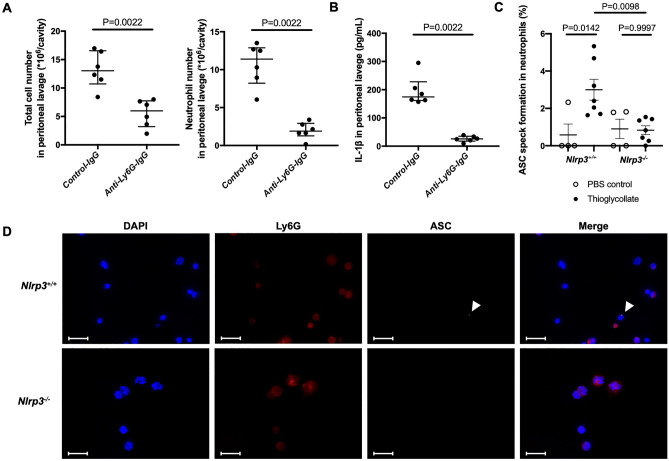
Neutrophils are required for IL-1β release and show signs of inflammasome activation (speck). (**A**, **B**) Thioglycollate-induced peritonitis model in wild-type mice pretreated with anti-Ly6G antibody or control antibody (n = 6:6), evaluation of lavage cells and fluid. (**A**) Total cell number, absolute numbers of neutrophils, and (**B**) IL-1β concentration in peritoneal lavage fluid. (**C**, **D**) *Nlrp3*^+*/*+^ and *Nlrp3*^*−/−*^ mice were i.p. injected with 1 mL sterile 3% thioglycollate broth (closed circles) or 1 mL sterile PBS (open circles). ASC speck formation in intraperitoneal cells was examined one hour afterward. (**C**) Quantification of neutrophils presenting ASC speck in a separate experiment (PBS control, n = 4:4; Thioglycollate, n = 7:7). (**D**) Representative microscopy images of immunostained cells in the intraperitoneal lavage fluid captured at 100x. Blue, DNA (DAPI); red, Ly-6G; green, ASC antibody staining. White arrows indicate ASC speck. Scale bar equals 20 µm.

To confirm that neutrophils are the predominant cells in our current model of thioglycollate-induced peritonitis, at 4 h^6^ we performed flow cytometry to distinguish neutrophils from macrophages in peritoneal fluid lavage. Ly6G-positive cells and F4/80-positive cells were defined as neutrophils and macrophages, respectively (representative gating is shown in Supplementary Fig. 1). Indeed, neutrophils were the predominant cells (more than 90% of total cells) in the peritoneal cavity 4 h after thioglycollate injection (Fig. 1A). To assess the role of NLRP3 in neutrophil-mediated inflammation, we induced peritonitis in both *Nlrp3*^*−/−*^ mice and *Nlrp3*^+*/*+^ mice. We observed 40% reduction in peritoneal cell infiltration in *Nlrp3*^*-/-*^ mice as compared to *Nlrp3*^+*/*+^ mice 4 h after thioglycollate injection (Fig. 1B). The reduction was in neutrophils while there were no differences in macrophages between the two genotypes (Fig. 1C). Since NLRP3 induces IL-1β release through inflammasome assembly also observed in neutrophils^3^, we measured the level of IL-1β in the peritoneal lavage fluid. Thioglycollate caused release of IL-1β in the peritoneum and this was highly NLRP3-dependent (Fig. 1D). To assess the direct contribution of neutrophils to the production of IL-1β, we induced thioglycollate-induced peritonitis in neutrophil-depleted mice using anti-Ly6G antibody. Neutrophils in the peritoneal fluid lavage were identified by Wright-Giemsa stain. Anti-Ly6G antibody reduced the total cell number and the absolute number of neutrophils in the peritoneal lavage fluid (Fig. 2A). The IL-1β concentration was significantly decreased in neutrophil-depleted mice (Fig. 2B). In addition, we evaluated inflammasome activation in the neutrophils recruited into the peritoneal cavity in the thioglycollate treated mice by visualizing ASC filaments, i.e. speck formation by immunofluorescence staining. Since neutrophil speck is short-lived after neutrophil stimulation^7^ we collected the lavage 1 h after thioglycollate stimulation (Fig. 2C). *Nlrp3*^+*/*+^ mice had a higher percentage of ASC speck positive neutrophils one hour after the thioglycollate challenge than *Nlrp3*^*−/−*^ mice (Fig. 2C and D).

**Figure 3:**
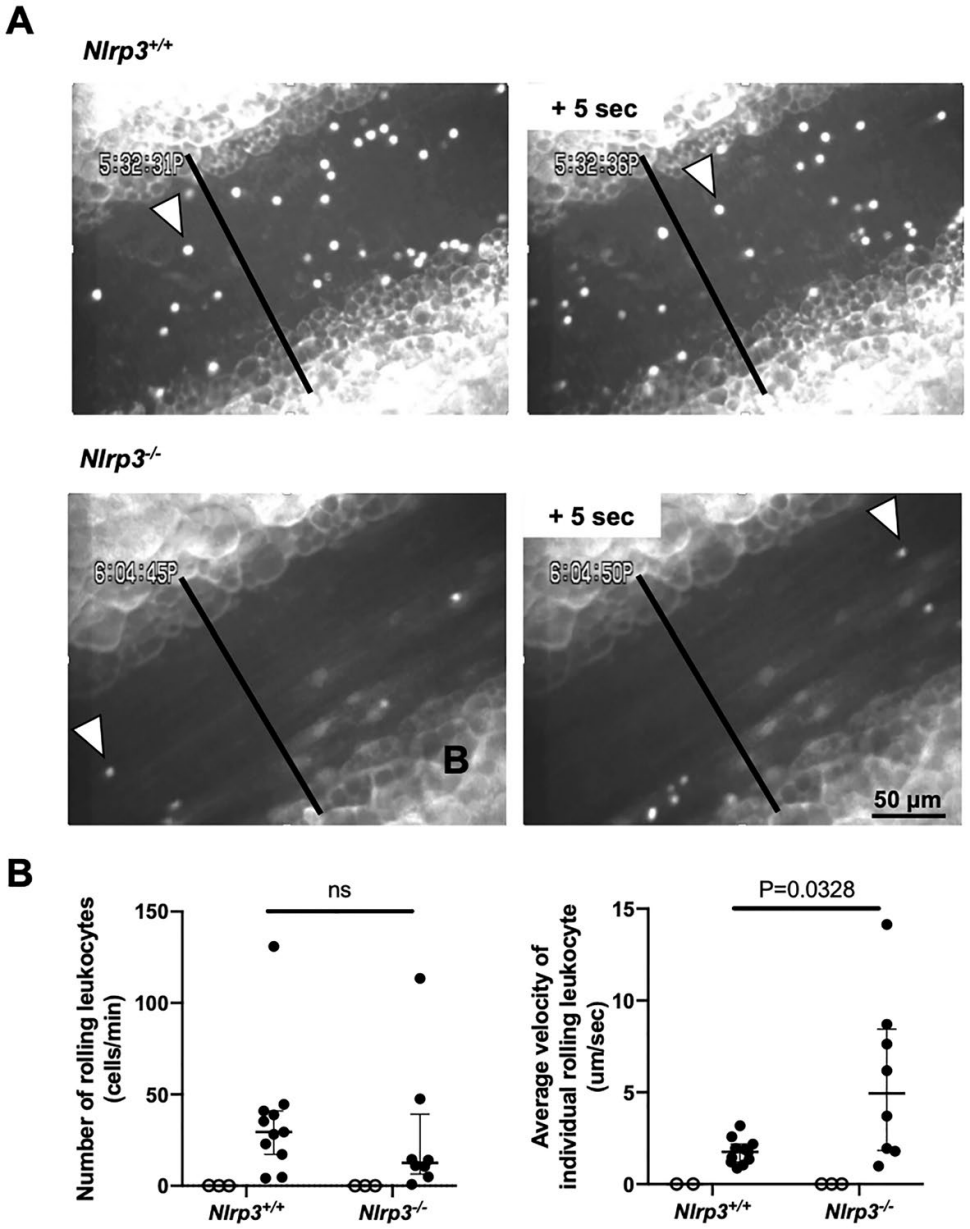
NLRP3 regulates velocity of leukocytes rolling on the inflamed vessel walls. Intravital microscopy of mesenteric venules. (**A**) representative intravital microscopy images from a *Nlrp3*^+*/*+^ (top) and *Nlrp3*^*−/−*^ (bottom) mouse treated with thioglycollate i.p. injection. All rolling leukocytes crossing the black vertical line through the mesenteric venule were counted. The white arrowheads points to the same leukocyte at 5 s interval. Supplementary videos are provided. (**B**) The number of rolling leukocytes on the vessel wall and the leukocyte velocity with or without thioglycollate treatment (PBS control, open circles, n = 3:3; Thioglycollate, closed circles, n = 11:8).

Based on these results, we hypothesized that neutrophil recruitment was reduced in *Nlrp3*^*−/−*^ mice by the observed decreased production of IL-1β, a known activator of endothelial cells^8^. We then sought to evaluate endothelial activation supporting the critical step in leukocyte extravasation, i.e., leukocyte rolling in the mesenteric venules. *Nlrp3*^*−/−*^ mice showed higher leukocyte velocity as compared to *Nlrp3*^+*/*+^ venules (Fig. 3). There were no differences in numbers of rolling leukocytes per minute on the vessel wall between the groups as observed by intravital microscopy (Fig. 3A and B, and Supplementary Videos), indicating a difference in the strength of leukocyte-endothelial interaction resulting in neutrophils rolling with decreased velocity in *Nlrp3*^+*/*+^ mice compared to *Nlrp3*^*−/−*^ mice.

**Figure 4:**
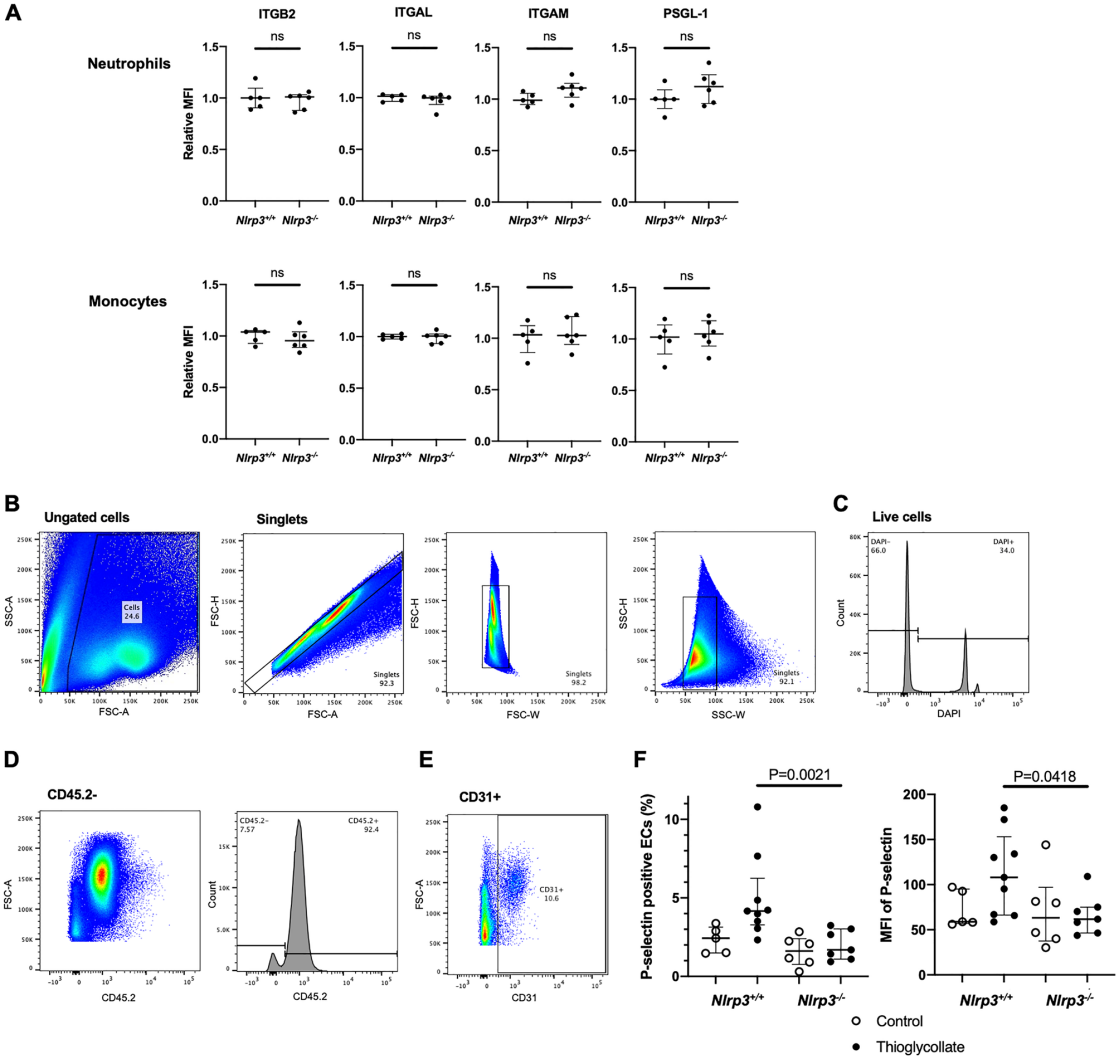
*Nlrp3* deficiency reduces membrane expression of P-selectin on the inflamed endothelial cells, while circulating leukocyte adhesion receptors expression is not altered. Neutrophils and monocytes in peripheral blood were collected from *Nlrp3*^+*/*+^ and *Nlrp3*^*−/−*^ mice 4 h after injection of thioglycollate broth (n = 5:6). (**A**) Surface expressions of integrins; integrin β2 (ITGB2, CD18), integrin αL (ITGAL, CD11a), integrin αM (ITGAM, CD11b), and P‐selectin glycoprotein ligand 1 (PSGL-1, CD162) were analyzed by flow cytometry. The relative MFI was calculated as a relative value to an average MFI of *Nlrp3*^+*/*+^*.* (B-F) Endothelial cells were isolated from the mesentery of *Nlrp3*^+*/*+^ and *Nlrp3*^*−/−*^ mice 4 h after injection with thioglycollate (Thioglycollate, closed circles, n = 9:7) or PBS (Control, open circles, n = 5:6). Surface P-selectin expression was analyzed by flow cytometry. (**B**–**E**) Gating strategy for isolated endothelial cells. Endothelial cells were defined as negative for CD45.2 and positive for CD31; (**B**) ungated cells and singlets, (**C**) live cells as negative for DAPI, (**D**) CD45.2 negative and (**E**) CD31 positive. (**F**) P-selectin expression on gated endothelial cells. Left: percentage of P-selectin positive endothelial cells. Right: mean fluorescent intensity (MFI) of P-selectin.

To elucidate the mechanism of the faster leukocyte rolling in *Nlrp3*^*−/−*^ mice, we sought to evaluate the levels of neutrophil and endothelial adhesion molecules implicated in early inflammatory response. We used flow cytometry to determine the neutrophil surface expression of adhesion molecules, essential for leukocyte adhesion, including integrin β2 (ITGB2, CD18), integrin αL (ITGAL, CD11a), integrin αM (ITGAM, CD11b), and P‐selectin glycoprotein ligand 1 (PSGL-1, CD162) by flow cytometry. Neutrophils and monocytes both in peripheral blood and lavage fluid after the thioglycollate-induced peritonitis demonstrated no differences in these adhesion molecules (Fig. 4A and Supplementary Fig. 2A).

Knowing about the importance of endothelial adhesion molecules in leukocyte recruitment, we sought to evaluate the adhesion molecules known to be responsible for leukocyte rolling early after the onset of inflammation on the endothelial cells, i.e., P-selectin^9^. Endothelial cells were isolated from the mesentery of *Nlrp3*^*−/−*^ and *Nlrp3*^+*/*+^ mice 4 h after thioglycollate injection, and we compared their surface P-selectin expression by flow cytometry. The gating strategy for isolated endothelial cells is shown in Fig. 4B–E. Both percentages of P-selectin positive endothelial cells and mean fluorescent intensity of P-selectin were reduced in *Nlrp3*^*−/−*^ mice compared with *Nlrp3*^+*/*+^ mice (Fig. 4F). This indicates lower activation of endothelial cells obtained from *Nlrp3*^*−/−*^ mice.

## Discussion

**Figure 5:**
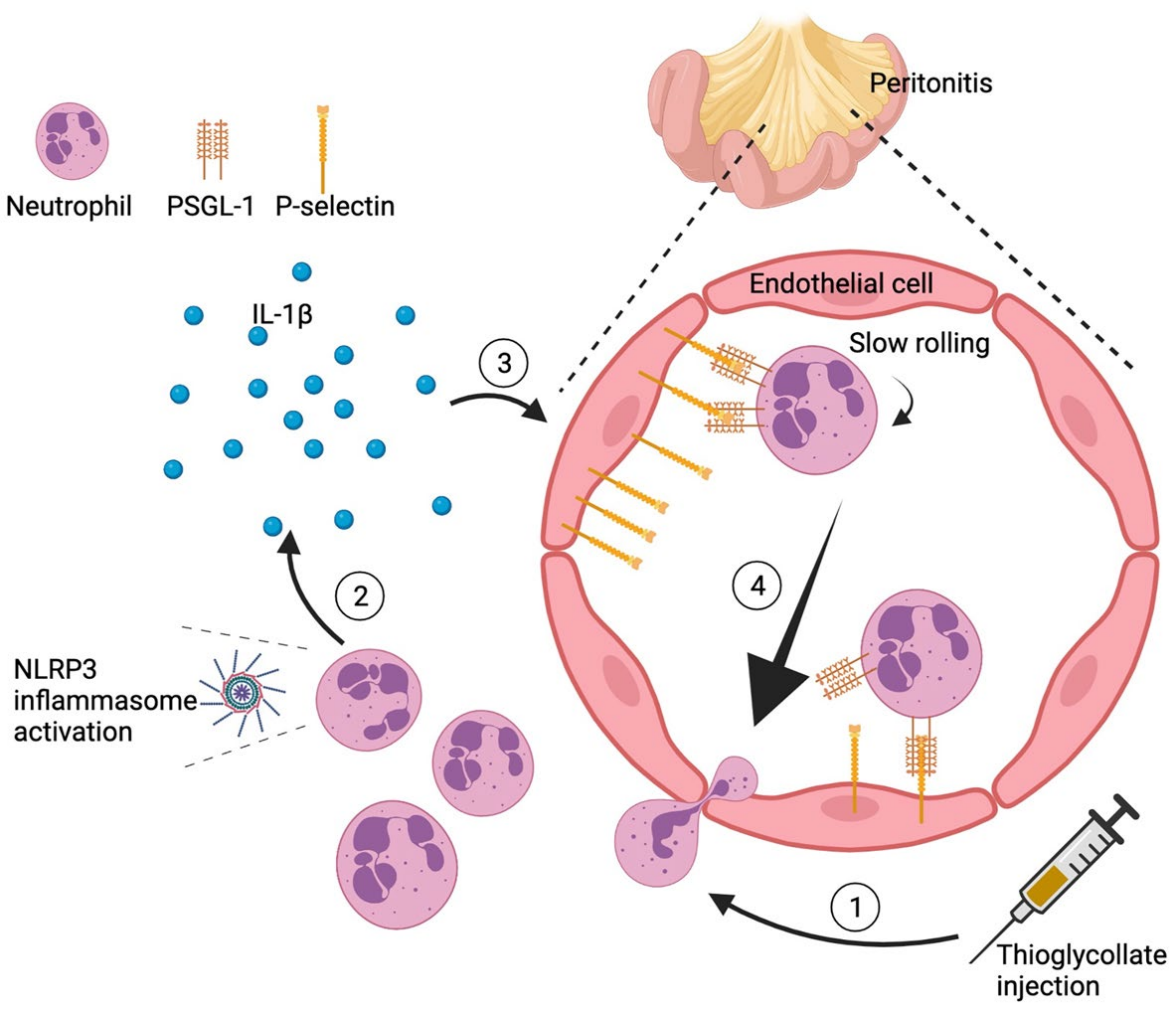
A likely interpretation of our results. 1: Thioglycollate injection initiates neutrophil-predominant peritonitis. 2: Neutrophils induce IL-1β production likely through NLRP3 inflammasome activation. 3: Released IL-1β activates endothelial cells leading to increased P-selectin expression. 4: Increased P-selectin expression results in slower neutrophil rolling and higher neutrophils extravasation.

Our study suggests an important role of *Nlrp3* and neutrophils in promoting endothelial cell activation during acute inflammation observed in the mouse thioglycollate-induced peritonitis model. *Nlrp3* deficiency reduced neutrophil recruitment and lowered IL-1β production in the inflamed peritoneal cavity. IL-1β was not generated when neutrophils were depleted. Since IL-1β regulates P-selectin expression^10,11^, NLRP3-deficiency also resulted in the lowering of surface expression of P-selectin on endothelial cells. The subsequent reduction of endothelial adhesiveness led to higher rolling velocities and the observed decrease in leukocyte transmigration in the *Nlrp3*^*-/-*^ animals. We propose a potential sequence of events leading to the thioglycollate-induced inflammatory response in Fig. 5.

The thioglycollate-induced peritonitis model (at 4 h) has been characterized as neutrophil-dominant inflammation^6,9^, and it is reasonable to speculate that the high number of neutrophils in the peritoneal cavity are the sources of the IL-1β. Neutrophil depletion by anti-Ly6G antibody indeed prominently reduced levels of IL-1β in peritoneal fluid. In addition, *Nlrp3*^+*/*+^ neutrophils, infiltrated in the peritoneal cavity at early stages (1 h) after thioglycollate induced peritonitis, showed inflammasome assembly as ASC speck formation. This result is consistent with our previous report in which the dynamic observation revealed ASC speck in neutrophils within an hour after stimulation^7^. Although other innate immune cells can assemble NLRP3 inflammasome^12^ our findings support the notion that neutrophils are the predominant source of IL-1β production early in inflammation. Our hypothesis that neutrophil NLRP3 inflammasome activation plays a primary role in thioglycollate response is consistent with the recent report documenting the importance of a gain-of-function mutation in *Nlrp3* specifically in neutrophils in autoinflammatory disease^13^. On the other hand, NLRP3 possibly regulates inflammation through other neutrophil functions. In 2012, NLRP3 inflammasome was reported to promote chemotactic immune cell migration to the CNS in experimental autoimmune encephalomyelitis (EAE), an animal model of multiple sclerosis^14^. NLRP3 inflammasome upregulated chemotaxis/migration-related proteins in the immune cells and adhesion receptors. We have recently observed that in neutrophils, similar to macrophages, NLRP3 inflammasome assembles at the microtubule organizing center^7^. Thus, NLRP3 inflammasome could also be implicated in neutrophil movement, a possibility we are currently addressing. The potential role of NLRP3 in the activities of phagocytes has also been reported in both septic and aseptic conditions. NLRP3 depletion enhanced neutrophil phagocytosis activity and bacterial clearance in a polymicrobial sepsis mouse model^15^ and ameliorated the impaired phagocytosis in oxygen-glucose-deprived BV-2 microglial cells as the condition of post-ischemic stroke^16^.

The reduced endothelial plasma membrane P-selectin expression in *Nlrp3*-deficient mice after thioglycollate injection is of interest. P-selectin is stored in Weibel-Palade bodies of endothelial cells and is transported to the cell surface rapidly with activation of endothelial cells. Once expressed on the surface, P-selectin is again internalized for recycling inside the cell after 20–30 min^17,18^. In parallel, the transcription and translation of P-selectin occur independently if stimulation lasts several hours^19,20^. The surface expression of P-selectin is seen as biphasic and enhanced time-dependently in several in vivo models^21–23^. IL-1β is known to act directly on endothelial cells thereby affecting the adhesiveness of the endothelial cell surface for neutrophils^8^. Importantly, previous reports showed that IL-1β treatment increased surface expression of P-selectin at 4 h in vivo and at 1.5 h in vitro on mouse primary endothelial cells^10,11^. Given this information, it is reasonable to assume that IL-1β released at the inflammatory site through the activation of NLRP3 inflammasomes stimulated endothelial cells leading to the sustained expression of P-selectin on their surface (Fig. 5).

Higher rolling velocities in *Nlrp3*-deficient mice are consistent with the reduced P-selectin expression in *Nlrp3*-deficient mice. Because lower rolling velocity favors leukocyte transmigration, P-selectin is essential in driving the inflammatory process through leukocyte recruitment^9^. We suggest that this reduced presence of P-selectin on the endothelial surface in *Nlrp3*^*-/-*^ mice is responsible for the reduced recruitment of leukocytes, with a specific focus on neutrophils in the thioglycollate-induced peritonitis model.

In conclusion, we demonstrated that *Nlrp3*-deficiency reduced neutrophil recruitment and caused lower levels of IL-1β in the inflamed peritoneal cavity. Furthermore, we showed that *Nlrp3* deficiency decreased the expression of adhesion molecules on endothelial cells, but not on leukocytes, leading to higher velocities of leukocyte rolling at the site of inflammation and consequently lower leukocyte transmigration. Together, these data suggest that NLRP3 regulates neutrophil dominant acute inflammation through IL-1β production, controlling endothelial cell activation, and neutrophil transmigration towards the inflammatory irritant.

## Methods

A detailed list of materials used and corresponding ordering information can be found in the Supplementary Information.

### Accordance statement

We confirm that all methods were performed in accordance with the ARRIVE (Animal Research: Reporting of In Vivo Experiments) guidelines^24^ and relevant guidelines.

### Animals

*Nlrp3*^*−/−*^ (stock no. #021,302) and corresponding wild-type (C57BL/6 J; stock no. #000,664) mice were obtained from Jackson Laboratory (Bar Harbor, ME, USA) and bred in-house. Mouse lines were housed in the animal facility of Boston Children’s Hospital. All animals were housed in accordance with the institutional animal facility, and mice of both sexes were randomly assigned for experiments. Each experiment had n = 3–6 mice/control group and n = 7–11 mice/treated group. Data analysis was blinded to the identity of the sample. The experimental animal procedures in this study were approved by the Institutional Animal Care and Use Committee of Boston Children’s Hospital under the protocol numbers 20- 01-4096R and 20-02-4097R in accordance with the ARRIVE guidelines, and all the experiments were carried out in compliance with the relevant guidelines and regulations.

### Thioglycollate-induced peritonitis model

Experiments were performed according to a previously described protocol^25^ with some modifications. Thioglycollate medium was dissolved in distilled water in 3% concentration, autoclaved, and aged for at least one month, avoiding light at room temperature. Female and male 8–10-week-old mice were administered 1 ml thioglycollate broth intraperitoneally. At 4 h after initiation, mice were euthanized, and cells in the peritoneal cavity were harvested through injection of an additional 4 mL of sterile ice-cold phosphate buffered saline (PBS) containing 2 mM ethylenediaminetetraacetic acid (EDTA) as peritoneal lavage with gentle massaging of the peritoneal wall. The tota1 cell numbers were determined in a hemocytometer. From the total cell count in the peritoneal lavage fluid, the percentage of neutrophils or macrophages was determined by flow cytometry, and the absolute number of neutrophils and macrophages in the peritoneal lavage was calculated.

### Depletion of neutrophils

Neutrophil depletion was performed by intravenous injection of 5 μg/g mouse of rat anti-Ly-6G antibody retro-orbitally. Control antibody (5 μg/g mouse) was injected in control mice. Thioglycollate was administered 18 h after injection of the antibodies.

### Immunofluorescence staining of ASC speck in neutrophils

At one hour after initiation of thioglycollate-induced peritonitis, mice were euthanized, and cells in the peritoneal cavity were harvested. The cells were fixed with 4% PFA for 1 h at room temperature (RT), washed once with PBS, permeabilized (0.1% Triton X-100, 0.1% sodium citrate) for 10 min at 4 °C, and incubated with blocking buffer (2.5% BSA, 0.5% Tween-20 in 1 × PBS) at 37 °C for 1 h. Samples were incubated at 4 °C with the primary antibodies against Ly-6G (2.5 μg/mL) and ASC (0.12 μg/mL) and subsequently washed 3 times with PBS before incubation with the secondary antibodies (1.33 μg/mL) for 2 h at RT. After another 3 washing steps with PBS, neutrophils were counterstained with Hoechst 33342 (1.0 µg/mL) and mounted using anti-fade fluorescent mounting medium. Images were acquired as Z-stacks of 0.1625 µm size and stitched together on a Keyence BZ-X810 microscope, using 60 × magnification. Each sample contained at least 100 neutrophils, and the percentage of neutrophils forming ASC speck, a readout for inflammasome activation, was quantified. Images were identically acquired and processed with Fiji/ImageJ.

### Endothelial cell isolation

We followed a preexisting protocol^26,27^ with some modifications. In brief, 4 h after the thioglycollate challenge, mice were perfused with ice-cold PBS transcardially until the colorless fluid was observed coming from the right atrium. The mesentery was collected, rinsed, and minced mechanically into cubes smaller than 1 mm. Following incubation with Accumax for 30 min at room temperature, the mesentery was filtered through a 70 μm cell strainer to obtain a single cell suspension. After centrifugation at 350 g for 5 min at 4 °C, the floating fat layer and the supernatant were aspirated. The cell pellet was resuspended in the desired amount of flow cytometry buffer (PBS with calcium and magnesium, 2% FBS) with 20 μg/mL DNase I and used for flow cytometric analysis.

### Intravital microscopy and image analysis

Experiments were performed according to our previously described protocol^28^ with some modifications. Briefly, 4–6 week old mice were anesthetized with 300 mg/kg of avertin and 50 μL of 1 mg/mL Rhodamine 6G was injected retro-orbitally to label circulating leukocytes. The mesentery was gently exteriorized through a midline abdominal incision. A mesenteric venule (150 ± 50 μm) was visualized with a Zeiss Axiovert 135 inverted microscope (objective: × 32, 0.4 n.a.) equipped with a 100-W HBO fluorescent lamp source with a narrow-band FITC filter set and a silicon-intensified tube camera connected to a DVD recorder. For quantification, venules were recorded for 5 min. The first and last minute were excluded, and 3 min in the middle were analyzed. The number of rolling leukocytes crossing the plane perpendicular to the venule axis were counted, and the average value per 1 min in the standardized diameter was calculated. The velocity of leukocyte rolling was determined by measuring the distance that a single leukocyte traveled and the required time using Fiji/ImageJ software. Approximately 25 leukocytes randomly chosen were measured, and the average velocity was calculated.

### Flow cytometry

For the identification of neutrophils and macrophages in the peritoneal lavage, we defined APC/Cy7-CD45.2^+^ PB-F4/80^-^ PE-Ly6G^+^ cells as neutrophils, and CD45.2^+^ F4/80^+^ Ly6G^-^ cells as macrophages (Supplementary Fig. 1A–C). Circulating neutrophils and monocytes in whole peripheral blood were gated as PE-CD115^-^ PB-Ly6G^+^ and CD115^+^ Ly6G^-^, respectively (Supplementary Fig. 1D and E). To quantify the surface expression levels of integrins on leukocytes, neutrophils, and monocytes (or macrophages) in whole peripheral blood and peritoneal lavage fluid cells were stained with FITC-CD11a, AF700-CD11b, AF647-CD18, and AF647-CD162. To determine primary endothelial cells from mesentery, cells were initially selected by size based on a forward scatter (FSC) and a side scatter (SSC). Live cells (DAPI^-^) were gated on singlets, ensuring doublet discrimination by a sequential gating by FSC-A vs. FSC-H, FSC-W vs. FSC-H, and SSC-W vs. SSC-H. Endothelial cells were defined as APC/Cy7-CD45.2^-^ APC-CD31^+^ cells and FITC conjugated anti-CD62P antibody was used for the assessment of endothelial cell activation. Experiments were performed using a BD LSRFortessa Flow Cytometer equipped with 3 lasers, and data was analyzed using FlowJo.

### Enzyme-linked immunosorbent assay (ELISA) for peritoneal lavage fluid

The peritoneal lavage fluid was concentrated using Amicon Ultra-0.5 mL 3 K centrifugal filters in accordance with the manufacturer’s instructions. The levels of IL-1β were determined with ELISA MAX™ Deluxe Set Mouse IL-1β in accordance with the manufacturer’s instructions.

### Statistics

Data were described with the median and interquartile range (IQR) for quantitative variables. We assessed the association between variables using Wilcoxon’s rank-sum test for quantitative variables. All tests were two-sided, and a *p*-value < 0.05 was considered significant. All statistical analyses were performed using GraphPad Prism ver. 7.0 (GraphPad Software, San Diego, CA, USA).

### Ethical approval

DDW is on the Scientific Advisory Board of Neutrolis, and a consultant to Takeda Pharmaceutical Company Limited.

## Supplementary Information

Supplementary Information 1.

Supplementary Video 1.

Supplementary Video 2.

## Data Availability

The datasets used and analyzed in this study are available from the corresponding authors on reasonable requests.

## References

[1] Wagner DD, Frenette PS (2008). The vessel wall and its interactions. Blood.

[2] Swanson KV, Deng M, Ting JPY (2019). The NLRP3 inflammasome: Molecular activation and regulation to therapeutics. Nat. Rev. Immunol..

[3] Münzer P (2021). NLRP3 Inflammasome assembly in neutrophils is supported by PAD4 and promotes NETosis under sterile conditions. Front. Immunol..

[4] Amaral FA (2012). NLRP3 inflammasome-mediated neutrophil recruitment and hypernociception depend on leukotriene B(4) in a murine model of gout. Arthritis Rheum..

[5] Inoue Y (2014). NLRP3 regulates neutrophil functions and contributes to hepatic ischemia-reperfusion injury independently of inflammasomes. J. Immunol..

[6] Frenette PS, Mayadas TN, Rayburn H, Hynes RO, Wagner DD (1996). Susceptibility to infection and altered hematopoiesis in mice deficient in both P- and E-selectins. Cell.

[7] Aymonnier K (2022). Inflammasome activation in neutrophils of patients with severe COVID-19. Blood Adv..

[8] Bevilacqua MP, Pober JS, Wheeler ME, Cotran RS, Gimbrone MA (1985). Interleukin-1 activation of vascular endothelium. Effects on procoagulant activity and leukocyte adhesion. Am. J. Pathol..

[9] Mayadas TN, Johnson RC, Rayburn H, Hynes RO, Wagner DD (1993). Leukocyte rolling and extravasation are severely compromised in P selectin-deficient mice. Cell.

[10] Harari OA (1999). Endothelial cell E- and P-selectin up-regulation in murine contact sensitivity is prolonged by distinct mechanisms occurring in sequence. J. Immunol..

[11] Villar IC (2011). Suppression of endothelial P-selectin expression contributes to reduced cell trafficking in females. Arterioscler. Thromb. Vasc. Biol..

[12] Franchi L, Eigenbrod T, Muñoz-Planillo R, Nuñez G (2009). The inflammasome: a caspase-1-activation platform that regulates immune responses and disease pathogenesis. Nat. Immunol..

[13] Stackowicz J (2021). Neutrophil-specific gain-of-function mutations in Nlrp3 promote development of cryopyrin-associated periodic syndrome. J. Exp. Med..

[14] Inoue M, Williams KL, Gunn MD, Shinohara ML (2012). NLRP3 inflammasome induces chemotactic immune cell migration to the CNS in experimental autoimmune encephalomyelitis. Proc. Natl. Acad. Sci. U. S. A..

[15] Jin L, Batra S, Jeyaseelan S (2017). Deletion of Nlrp3 augments survival during polymicrobial sepsis by decreasing autophagy and enhancing phagocytosis. J. Immunol..

[16] Schölwer I (2020). NLRP3 depletion fails to mitigate inflammation but restores diminished phagocytosis in BV-2 cells after in vitro hypoxia. Mol. Neurobiol..

[17] Subramaniam M, Koedam JA, Wagner DD (1993). Divergent fates of P-and E-selectins after their expression on the plasma membrane. Mol. Biol. Cell.

[18] Straley KS, Green SA (2000). Rapid transport of internalized P-selectin to late endosomes and the Tgn: Roles in regulating cell surface expression and recycling to secretory granules. J. Cell Biol..

[19] Hahne M, Jäger U, Isenmann S, Hallmann R, Vestweber D (1993). Five tumor necrosis factor-inducible cell adhesion mechanisms on the surface of mouse endothelioma cells mediate the binding of leukocytes. J. Cell Biol..

[20] Sanders WE, Wilson RW, Ballantyne CM, Beaudet AL (1992). Molecular cloning and analysis of in vivo expression of murine P-selectin. Blood.

[21] Zhang R, Chopp M, Zhang Z, Jiang N, Powers C (1998). The expression of P- and E-selectins in three models of middle cerebral artery occlusion. Brain Res..

[22] Akgür F, Zibari G, McDonald J, Granger D, Brown M (2000). Kinetics of P-selectin expression in regional vascular beds after resuscitation of hemorrhagic shock: a clue to the mechanism of multiple system organ failure. Shock.

[23] Eppihimer MJ, Wolitzky B, Anderson DC, Labow MA, Granger DN (1996). Heterogeneity of expression of E- and P-selectins in vivo. Circ. Res..

[24] du Sert NP (2020). The ARRIVE guidelines 2.0: Updated guidelines for reporting animal research. PLOS Biol..

[25] Bosse R, Vestweber D (1994). Only simultaneous blocking of the L- and P-selectin completely inhibits neutrophil migration into mouse peritoneum. Eur. J. Immunol..

[26] Crewe C (2018). An endothelial-to-adipocyte extracellular vesicle axis governed by metabolic state. Cell.

[27] Dumas SJ (2021). Protocols for endothelial cell isolation from mouse tissues: kidney, spleen, and testis. STAR Protoc..

[28] Frenette PS, Johnson RC, Hynes RO, Wagner DD (1995). Platelets roll on stimulated endothelium in vivo: An interaction mediated by endothelial P-selectin. Proc. Natl. Acad. Sci. U. S. A..

