# Supplementary material for: NLRP3 inflammasome activation in neutrophils directs early inflammatory response in murine peritonitis
Source: Sci Rep. 2022 Dec 9;12:21313. doi: 10.1038/s41598-022-25176-4 (PMC9734191; doi:10.1038/s41598-022-25176-4)
Supplement: Supplementary file 1 — Supplementary Information 1. [file 41598_2022_25176_MOESM1_ESM.docx]

*Article*

**NLRP3 inflammasome activation in neutrophils directs early inflammatory response in murine peritonitis**

Saeko Fukui^1^, Shoichi Fukui^1,2^, Stijn Van Bruggen^1,4^, Lai Shi^1^, Casey E Sheehy^1^**,** Long Chu^1^,
Denisa D Wagner^1,2,3*^

^1^Program in Cellular and Molecular Medicine, Boston Children's Hospital, Boston, MA 02115, USA.

^2^Department of Pediatrics, Harvard Medical School, Boston, MA 02115, USA.

^3^Division of Hematology/Oncology, Boston Children's Hospital, Boston, MA 02125, USA.

^4^Centre of Molecular and Vascular Biology, Department of cardiovascular sciences, KU Leuven, 3000 Leuven, Belgium.

***Correspondence:**

Denisa D Wagner

denisa.wagner@childrens.harvard.edu

**Supplementary Figures**

**Supplementary Figure 1. Gating strategy of neutrophils, monocytes, and macrophages from peripheral blood and peritoneal lavage fluid.**

**
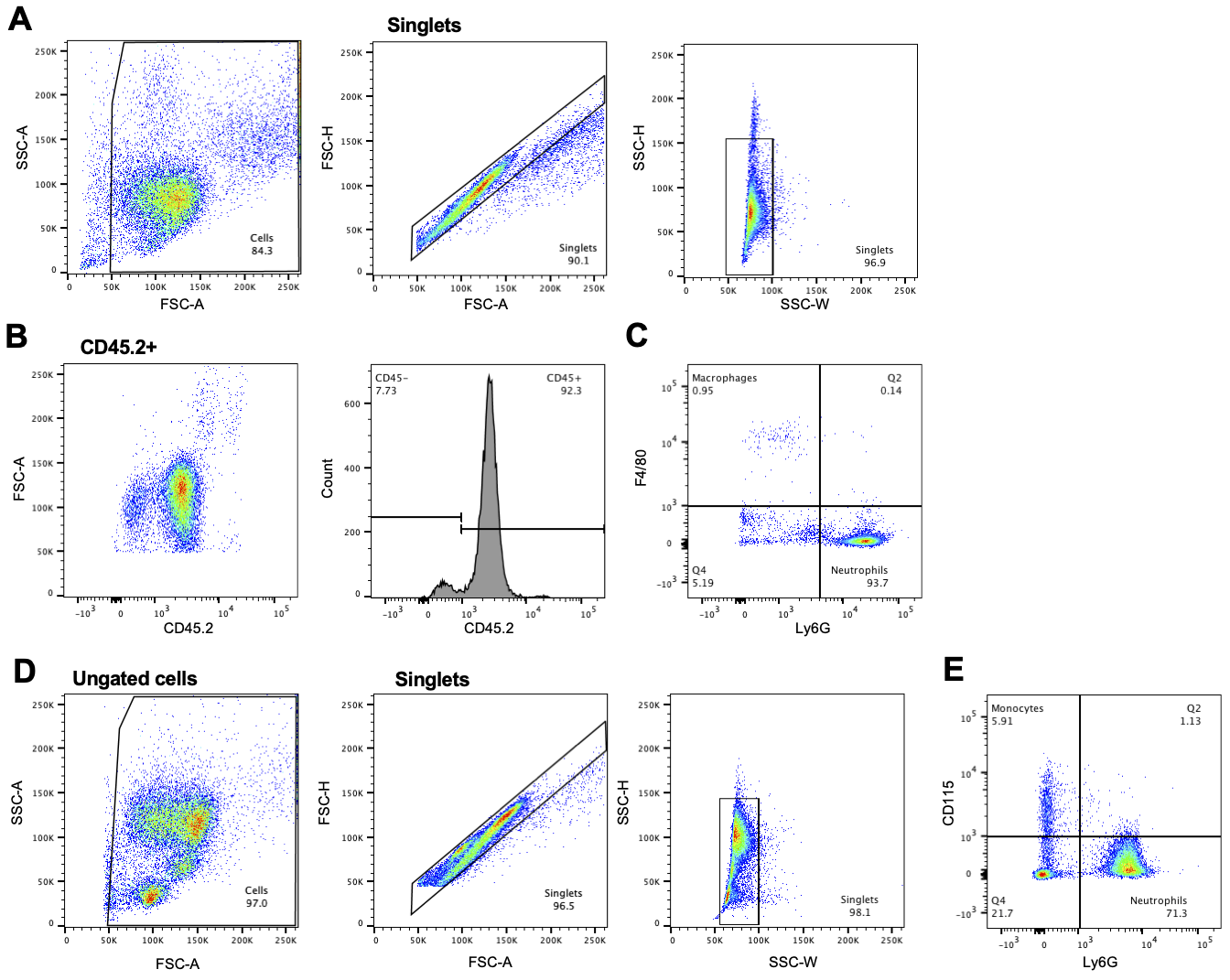
**

(A-C) Peritoneal lavage fluid. Neutrophils were defined as positive for CD45.2 and Ly6G, and macrophages were defined as positive for CD45.2 and F4/80. (A) Ungated cells and singlets, (B) CD45.2 positive, and (C) Ly6G or F4/80 positive. (D and E) Peripheral blood. Neutrophils and monocytes were defined as positive for Ly6G and positive for CD115, respectively. (D) Ungated cells and singlets, (E) Ly6G or CD115 positive.

**Supplementary Figure 2. *Nlrp3* does not change levels of adhesion molecules on leukocytes in the peritoneal cavity.**

**
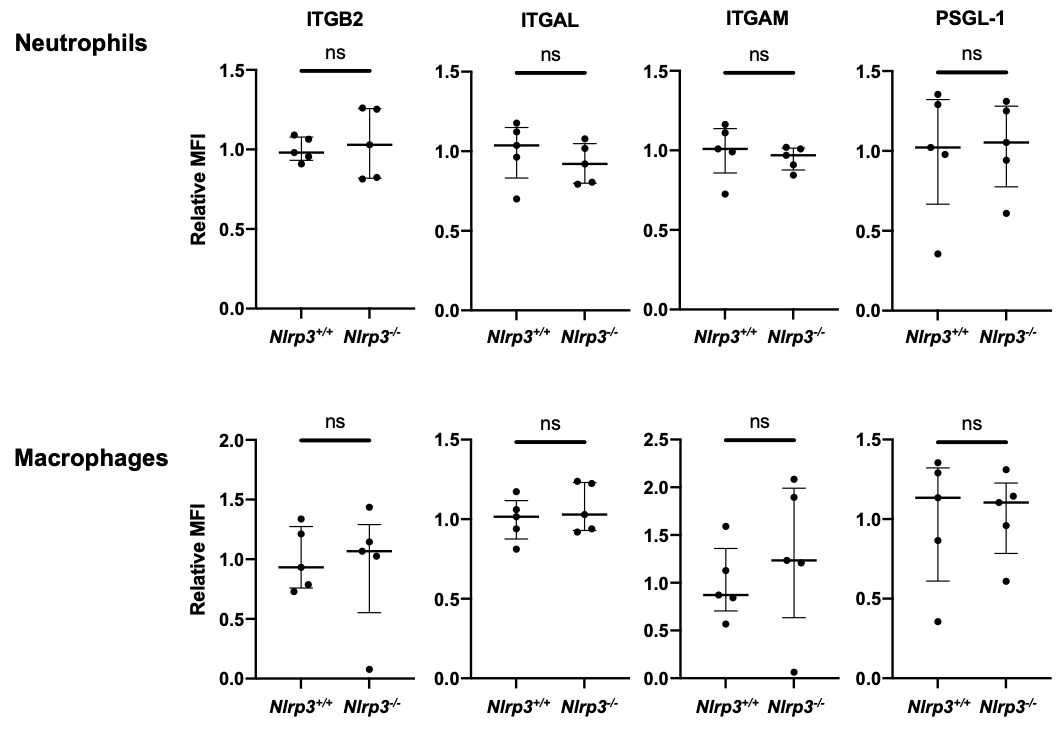
**

Peritoneal lavage fluid was collected from *Nlrp3^+/+^* and *Nlrp3^-/-^* mice 4 hours after with injections of thioglycollate broth. Surface expressions of integrins; integrin β2 (ITGB2, CD18), integrin αL (ITGAL, CD11a), integrin αM (ITGAM, CD11b), and P‐selectin glycoprotein ligand 1 (PSGL-1, CD162) were analyzed by flow cytometry. The relative mean fluorescent intensity (MFI) was calculated as a relative value to an average MFI of *Nlrp3^+/+^.* Neutrophils (top) and macrophages (bottom) in peritoneal lavage fluid (n=5:5).

**Supplementary Videos. Intravital microscopy.**

Representative videos of leukocyte rolling on the mesenteric vessel of a *Nlrp3^+/+^* and a *Nlrp3^-/-^* mouse 4 hours after injection of thioglycollate.

**Supplementary Tables. List of reagents and equipments**

**Supplementary Table S1. Thioglycollate-induced peritonitis model**

| Material | Catalog number | Company |
| --- | --- | --- |
| Thioglycollate medium | T9032 | Sigma-Aldrich |

**Supplementary Table S2. Immunofluorescence Staining of ASC Speck in Neutrophils**

| Material | clone | Catalog number | Company |
| --- | --- | --- | --- |
| Ultra-LEAF™ Purified anti-mouse  Ly-6G Antibody | 1A8 | 127632 | BioLegend |
| ASC/TMS1 Rabbit mAb (Mouse Specific) | D2W8U | 67824 | Cell Signaling Technology |
| Alexa Fluor™ 488  Donkey anti-Rabbit IgG | - | A-21206 | Invitrogen |
| Alexa Fluor™ 555  Goat anti-Rat IgG | - | A-21434 | Invitrogen |
| Hoechst 33342 | - | H3570 | Invitrogen |

**Supplementary Table S3. Depletion of Neutrophils**

| Material | clone | Catalog number | Company |
| --- | --- | --- | --- |
| Ultra-LEAF™ Purified anti-mouse  Ly-6G Antibody | 1A8 | 127632 | BioLegend |
| Ultra-LEAF™ Purified Rat IgG2a, κ | RTK2758 | 400544 | BioLegend |

**Supplementary Table S4. Endothelial cell isolation**

| Material | Catalog number | Company |
| --- | --- | --- |
| Accumax | AM-105 | Innovate Cell Technologies |
| Dnase I | #07469 | STEMCELL TECHNOLOGIES |

**Supplementary Table S5. Intravital microscopy and image analysis**

| Material or equipment | Catalog number | Company |
| --- | --- | --- |
| Avertin (2-tribromoethanol) | CAS: 75-80-9 | Sigma-Aldrich |
| Rhodamine 6G | CAS: 989-38-8 | Sigma-Aldrich |
| 100-W HBO fluorescent lamp source | - | Opti Quip |
| Narrow-band FITC filter set | - | Chroma Technology |
| Silicon-intensified tube camera | C2400 | Hamamatsu |
| DVD recorder | Sony DVO 1000MD | Sony Electronics |

**﻿ Supplementary Table S6. Flow cytometry**

| Antibody | Clone | Catalog number | Company |
| --- | --- | --- | --- |
| APC/Cyanine7 anti-mouse CD45.2 | 104 | 109824 | BioLegent |
| Pacific Blue™ anti-mouse F4/80 | BM8 | 123124 | BioLegent |
| PE anti-mouse Ly6G (Gr-1) | RB6-8C5 | 12-5931-82 | eBioscience |
| PE anti-mouse CD115 | AFS98 | 12-1152-81 | eBioscience |
| Pacific Blue™ anti-mouse Ly6G | 1A8 | 127612 | BioLegent |
| FITC anti-mouse CD11a | 2D7 | 561683 | BD Pharmingen |
| Alexa Fluor® 700 anti-mouse/human CD11b | M1/70 | 101222 | BioLegent |
| Alexa Fluor® 647 anti-mouse CD18 | M18/2 | 101414 | BioLegent |
| Alexa Fluor® 647 Rat Anti-Mouse CD162 | 2PH1 | 562806 | BD Pharmingen |
| APC anti-mouse CD31 | 390 | 102410 | BioLegend |
| FITC Anti-Mouse CD62P | RB40.34 | 553744 | BD Pharmingen |
| TruStain FcX™ (anti-mouse CD16/32) | 93 | 101320 | BioLegent |

| Isotype control | Clone | Catalog number | Company |
| --- | --- | --- | --- |
| APC/Cyanine7 Mouse IgG2a, κ | MOPC-173 | 400230 | BioLegend |
| Pacific Blue™ Rat IgG2a, κ | RTK2758 | 400527 | BioLegend |
| PE Rat IgG2b, κ | RTK4530 | 400608 | BioLegend |
| PE Rat IgG2a, κ | RTK2758 | 400508 | BioLegend |
| FITC Rat IgG2a, κ | RTK2758 | 400506 | BioLegend |
| Alexa Fluor® 700 Rat IgG2b, κ | RTK4530 | 400628 | BioLegend |
| Alexa Fluor® 647 Rat IgG2a, κ | RTK2758 | 400526 | BioLegend |
| Alexa Fluor® 647 Rat IgG1, κ | RTK2071 | 400418 | BioLegend |
| APC Rat IgG2a, κ | RTK2758 | 400511 | BioLegend |
| FITC Rat IgG1, λ | G0114F7 | 401913 | BioLegend |

**Supplementary Table S7. Enzyme-linked immunosorbent assay (ELISA) in peritoneal lavage fluid**

| Material | Catalog number | Company |
| --- | --- | --- |
| Amicon Ultra-0.5 Centrifugal Filter Unit - 3kDa nominal molecular weight cutoff | UFC5003 | Millipore |
| ELISA MAX™ Deluxe Set Mouse IL-1β | 402604 | BioLegend |
